# A lymph node mediastinal foreign body reaction mimicking nodal metastasis: A case series

**DOI:** 10.3389/fmed.2022.1014617

**Published:** 2022-09-23

**Authors:** Lina Zuccatosta, Maria Agnese Latini, Federico Mei, Martina Bonifazi, Emanuela Barisione, Mario Salio, Stefano Gasparini, Francesca Gonnelli

**Affiliations:** ^1^Pulmonary Diseases Unit, Azienda Ospedali Riuniti, Ancona, Italy; ^2^Polytechnic University of Marche, Ancona, Italy; ^3^Pulmonary Diseases Unit, Ospedale San Martino, Genoa, Italy; ^4^Pulmonary Diseases Unit, Ospedale Ss. Antonio e Biagio e Cesare Arrigo, Alessandria, Italy

**Keywords:** EBUS-TBNA, lymph nodes enlargement, oxidized cellulose, lung cancer, case series

## Abstract

**Introduction:**

In the last decades, many haemostatic substances included oxidized cellulose topically applied have been used during surgery and their use have become a common practice. Oxidized cellulose (OC) is one of the most used haemostatic substances. However, different studies have shown the persistence of OC deposits after surgical procedures that may simulate recurrent malignancies and abscesses. We present a case series of patients with enlarged on CT and PET-FDG positive lymphadenopathies due to foreign body inflammatory reaction to OC after lung surgery for pulmonary malignancies.

**Methods:**

Retrospective chart review of patients from 2021 to 2022 who underwent EBUS-TBNA for the characterization of hilar and/or mediastinal lymphadenopathies and a histopathological diagnosis of foreign body inflammatory reaction to OC.

**Results:**

*Eight* patients were referred to “Ospedali Riuniti di Ancona” (*n* = 7) and “Ospedale San Martino” (Genoa) (*n* = 1) Interventional Pulmonology Units for the characterization of hilar and/or mediastinal lymphadenopathies. All the evaluated patients underwent surgical procedures for lung cancers within the previous 12 months. EBUS-TBNA was performed in all the patients to rule out nodal metastasis. The cyto-pathological evaluation revealed amorphous acellular eosinofilic material surrounded by inflammatory reaction. As no other apparent causes might explain this finding and considering the temporal relationship between the lymphadenopathy and the lung surgery, foreign body inflammatory reaction to OC is the most likely cause of the phenomenon.

**Conclusion:**

In patients who underwent surgery for lung cancer, especially within few months, the development of lymph node foreign body reaction due to surgical material retention should always be considered.

## Introduction

The role of ultrasound-guided transbronchial needle aspiration (EBUS-TBNA) in the diagnosis of hilar and mediastinal lymphadenopathies is well-known: in particular, it represents an excellent tool both in the diagnosis and in the staging of lung cancer (1).

International Guidelines suggest EBUS-TBNA as first step in ruling in and out lymph node (LN) metastasis in patients with lung cancer in case of hilar and/or mediastinal LN enlargement on Computed Tomography (CT) and/or fluoro-dessossi-glucose positron emission tomography (FDG-PET) positive lymphadenopathies (2, 3).

In the last decades, many topical hemostatic agents have been used during surgery and their use have become a common practice (4, 5). One of these substances is oxidized cellulose (OC), an absorbable passive hemostatic agent (6). It adsorbs blood and forms a gelatinous mass providing a strong matrix for platelet adhesion and clot formation (7). Its low pH also causes localized vasoconstriction, further enhancing the hemostatic effect. In addition, the low pH exhibits antibacterial properties, minimizing the risk of infection. It is extracted from natural alpha-grade cotton and is a 100% biodegradable organic material (8). Theoretically, the OC completely degrades into oligosaccharides. However, different studies and data have shown the persistence of OC deposits after surgical procedures and the presence of OC in LNs adjacent to the surgical field, that may simulate recurrent malignancies and abscesses (7, 9, 10).

Herein, we present a case series of patients with enlarged LNs on CT and PET-FDG positive lymphadenopathies due to a foreign body inflammatory reaction to OC after lung surgery for pulmonary malignancies.

## Methods

We performed a retrospective chart review of patients from 1st of January 2021 to 1st of January 2022 who underwent EBUS-TBNA for CT enlarged LNs and PET-FDG positive lymphadenopathies after lung surgery for cancer across two Italian hospitals (Azienda Ospedali Riuniti, Ancona; San Martino Hospital, Genoa). The cases in which histopathological diagnosis of foreign body inflammatory reaction to OC were selected and described in this series.

All patients signed written informed consent for the use of the anonymized data for research.

## Results

We report on eight patients who underwent Video-Assisted Thoracic Surgery (VATS) lobectomy (*n* = 7) or wedge resection (*n* = 1) for lung cancer between the 1^st^ of January 2021 and the 1st of January 2022. VATS right upper lobectomy was performed in 6 (75%) patients, 1 (12.5%) patient underwent VATS middle lobectomy, and 1 (12.5%) patient underwent VATS right upper wedge resection. All patients were enrolled in a post-surgical follow-up programme with CT of thorax and abdomen.

The mean age of the patients was 69 years. Five (62%) were previous smokers. Table 1 reports the main demographic, clinical, imaging and histopathological data of the enrolled patients.

**Table 1:** Demographic, clinical, imaging, and histopathological data of the patients included in this case series.

**Pre-operative data**		
Age (median, interquartile range)	69, 56–80	
Sex (*n*, %)	M (6, 75%)	
	F (2, 25%)	
Caucasian ethnicity (*n*, %)	Caucasian (8, 100%)	
Smoking (*n*, %)	Current smoker	0
	Past smoker	(5, 62%)
	Non-smoker	(3, 38%)
Histology of the lung cancer (*n*, %)	Squamous cell carcinoma	(1, 12%)
	Adenocarcinoma	(7, 88%)
**Follow-up data**		
Size of lymph nodes on CT scan (median, interquartile range) [*n* = 8]	2 cm	1, 5–2, 5 cm
SUVmax on FDG-PET (median, interquartile range) [*n* = 8]	3, 5	1, 3–12

CT, computed tomography; FDG-PET, fluoro-dessossi-glucose positrone emission tomography. Values in square brackets refer to the number of patients with available data.

**Figure 1:**
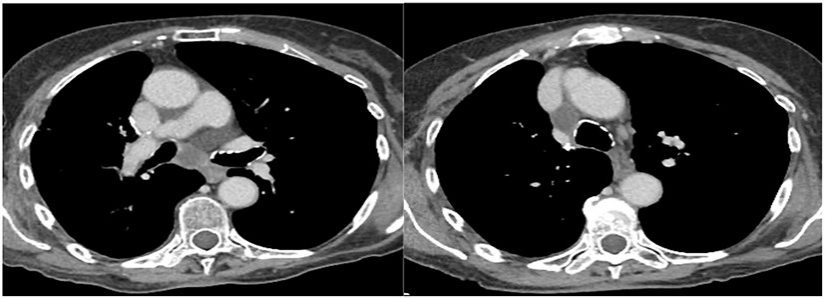
Subcarinal (7) and right inferior paratracheal (4R) lymphadenomegaly.

**Figure 2:**
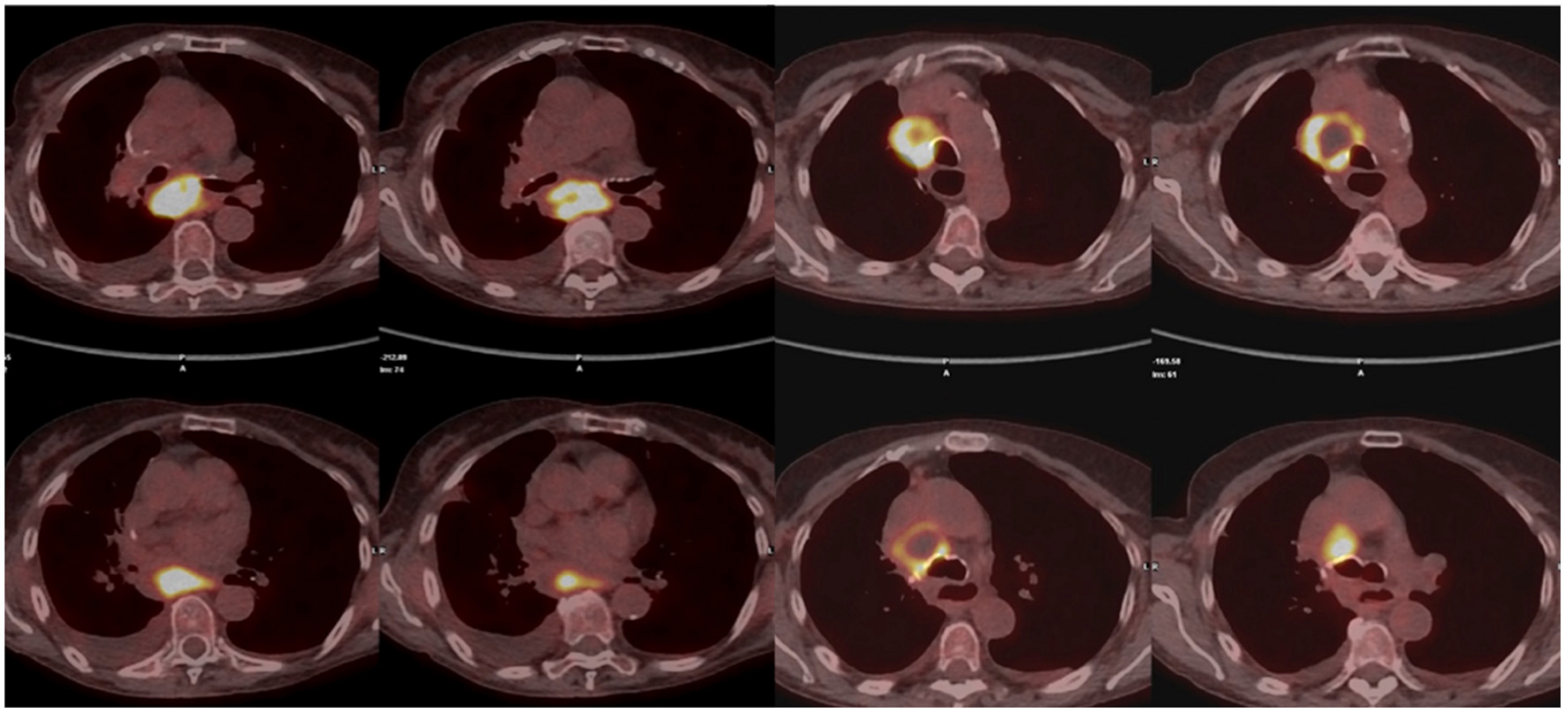
Subcarinal (7) and right inferior paratracheal (4R) FDG-PET positive lymph node.

During follow-up, all these patients were referred to “Ospedali Riuniti di Ancona” (*n* = 7) and “Ospedale San Martino” (Genoa) (*n* = 1) Interventional Pulmonology Units for the characterization of new onset hilar and/or mediastinal lymphadenopathies detected by imaging with CT LN enlargement (Figure 1) and intense metabolic activity on PET/CT (Figure 2).

All patients underwent surgical procedures for lung cancers within the previous 12 months (median 7 months, interquartile range 4–12 months).

Pathological sampling of suspicious LNs was performed by EBUS-TBNA in all patients. Sampled LNs were: right inferior paratracheal (4R) (*n* = 7), subcarinal (7) (*n* = 4), left inferior paratracheal (4L) (*n* = 1), and right hilar (11R) (*n* = 1) (in five patients more than one LN station was sampled).

**Figure 3:**
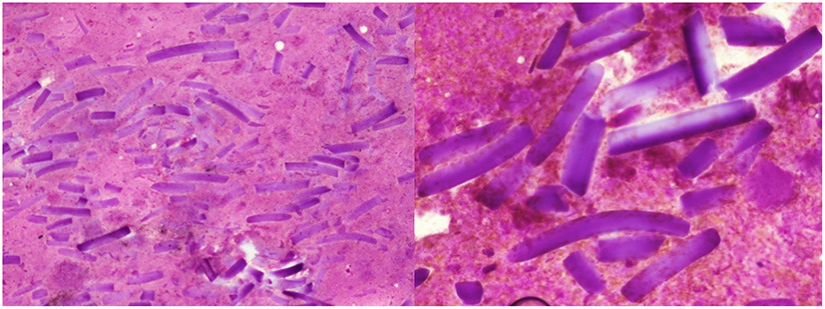
Histologic appearance: eosinophilic amorphous exogen material.

The cyto-pathological evaluation did not reveal atypical cells, rather showing amorphous acellular eosinofilic material surrounded by an inflammatory reaction (Figure 3) in all the cases.

The acellular component resulted to be part of the oxidized regenerated cellulose product (“*Tabotamp*^®^*, Ethicon SARL, Switzerland*”) used to obtain bleeding control during the previous chest surgery.

The histological findings described in the present case series can be included in the spectrum of the “foreign body reaction against biomaterials:” in 6 patients there was a chronic inflammation characterized mainly by macrophages, and in other two patients there were giant cells, the hallmark of foreign body reaction against biomaterials (11).

Finally, to estimate the prevalence of OC adverse reactions in lung surgery, we reviewed the register of lung cancer surgery at the “Ospedali Riuniti di Ancona.” A total of 118 patients underwent VATS for lung cancer between the 1st of January 2021 and the 1st of January 2022. OC was the only topical hemostatic agent used during mediastinal lymphadenectomy in that period in all patients. Therefore, we can estimate that the annual prevalence of OC adverse events was 5.9 events/100 person-years.

## Discussion

This case series reports 8 cases of foreign body inflammatory reaction in mediastinal LNs occurred soon after lung cancer surgery: mediastinal lymphadenopathies at FDG-PET/chest CT scan were found within the first year of follow-up (range 4–12 months) and EBUS-TBNA was performed in all the cases, ruling out cancer recurrence and showing amorphous exogenous material surrounded by inflammatory reaction.

No other apparent causes and/or interventions could explain this finding and considering the temporal relationship between the LN pathology and the use of the OC during lung surgery, foreign body inflammatory reaction to OC is the most likely cause of the phenomenon.

PET-CT scan is an excellent tool for the clinical diagnosis and staging of lung neoplasms and it plays an important role in the follow-up of patients to identify relapse of the disease. However, the possibility of benign conditions with inflammatory activity that may induce a false-positive high SUV on FDG-PET should always be considered also in cancer patients (12). The appearance of a new hypermetabolic lymphadenopathy during a lung cancer follow-up is not always synonymous of recurrence, so that the histological confirmation should be considered mandatory to establish the best therapeutic strategy.

In patients who underwent surgery for lung cancer, the development of LN foreign body reaction due to surgical material retention, even if rare, should always be considered (7, 13, 14).

OC is expected to be completely adsorbable (8) and should disappear from tissue in a maximum of 4 weeks; however, several studies report the persistency of this material causing foreign body reactions mimicking cancer or infection (5, 7, 13–16).

As summarized by Klopfleisch and Jung (11), the spectrum of foreign body against biomaterials can be highly variable: in some patients, there is a chronic inflammation characterized by macrophages, in some others, there are giant cells. Badanes et al. reported a clear granulomatous reaction (14), whereas Haidari et al. described an “high number of macrophages” (7); finally, in our cases the histopathological examination revealed eosinophilic amorphous material surrounded by macrophages and giant cells. All these histological features can be included in the spectrum of foreign body inflammatory reaction against biomaterials (11).

Since its introduction in use, cases that simulate tumor recurrence have been described in neurosurgery, gastrointestinal, and cardiac surgery (5, 15, 16), forcing additional examinations to determine the definite diagnosis of foreign body inflammatory reaction to biomaterials.

Cellulose fibers retention could be localized nearby their original position and could be the results of their anomalous degradation with consequent foreign body lymphadenitis.

Of note, we observed a foreign body inflammatory reaction within 12 months of follow-up, with a median of 7 months with a minimum of 4 months and a maximum of 12 months: the short latency between the surgical procedure and the onset of the lymphadenopathies should prompt pulmonologists to consider even other diagnostic hypothesis than cancer recurrence.

Moreover, Haidari et al. reported 7.5% of false positive diagnosis of cancer recurrence on CT scan follow-up due to OC material *(“Gelita-cel*^®^*, Gelita Medical, Germany”)* retention (7). Such a prevalence is similar to the one reported in this paper.

This case series demonstrates that the risk of incomplete degradation of OC components is relevant even with materials produced by other companies (i.e., “*Tabotamp*^®^*, Ethicon SARL, Switzerland*”), suggesting the need of limitation of their use in bleeding control during thoracic surgery.

This case series also reiterates the need to obtain cytohistological confirmation of PET-FDG positive LNs in cancer patients and further underline the essential role of EBUS-TBNA in the diagnostic work-up and follow-up of lung cancer.

## Conclusion

OC (“*Tabotamp*” in our cases) is a well-known excellent tool used to reduce bleeding in surgery and it is commonly used also in thoracic surgery. Even though a complete adsorption is expected within a few weeks, sometimes abnormal persistency of this material can occur, causing foreign body inflammatory reaction.

In patients who underwent surgery for lung cancer, especially within a few months, the development of LN foreign body reaction due to surgical material retention should always be considered.

This case series supports the importance of histological confirmation in case of hilar and/or mediastinal lymphadenopathies during lung cancer follow-up, since inflammatory reactions mimicking cancer recurrences, even if not frequently, can occur.

FDG-PET is not optimal to distinguish inflammation from cancer recurrence due to its relatively low specificity, whereas EBUS-TBNA seems to be an excellent tool to guide lung cancer follow-up and management in these cases.

## Data availability statement

The original contributions presented in the study are included in the article/supplementary material, further inquiries can be directed to the corresponding author.

## Ethics statement

Ethical review and approval was not required for the study on human participants in accordance with the local legislation and institutional requirements. The patients/participants provided their written informed consent to participate in this study.

## Author contributions

FG, LZ, and SG contributed substantially to the conception, design of the work, and drafted the work. All authors contributed to the acquisition, analysis, interpretation of data for the work, revised it critically for important intellectual content, provided approval for publication of the content, and agreed to be accountable for all aspects of the work in ensuring that questions related to the accuracy or integrity of any part of the work are appropriately investigated and resolved. All authors contributed to the article and approved the submitted version.

## Conflict of interest

The authors declare that the research was conducted in the absence of any commercial or financial relationships that could be construed as a potential conflict of interest. The reviewer RT declared a past co-authorship with the authors MS, SG, FM, and LZ to the handling editor.

## Publisher's note

All claims expressed in this article are solely those of the authors and do not necessarily represent those of their affiliated organizations, or those of the publisher, the editors and the reviewers. Any product that may be evaluated in this article, or claim that may be made by its manufacturer, is not guaranteed or endorsed by the publisher.
